# Quantitative imaging: systematic review of perfusion/flow phantoms

**DOI:** 10.1186/s41747-019-0133-2

**Published:** 2020-03-04

**Authors:** Marije E. Kamphuis, Marcel J. W. Greuter, Riemer H. J. A. Slart, Cornelis H. Slump

**Affiliations:** 1 Multimodality Medical Imaging M3i Group, Faculty of Science and Technology, Technical Medical Centre, University of Twente, PO Box 217, Enschede, The Netherlands; 2 Robotics and Mechatronics Group, Faculty of Electrical Engineering, Mathematics, and Computer Science, Technical Medical Centre, University of Twente, Enschede, The Netherlands; 3 Medical Imaging Center, Department of Nuclear Medicine and Molecular Imaging, University Medical Center Groningen, University of Groningen, Groningen, The Netherlands; 4 Biomedical Photonic Imaging Group, Faculty of Science and Technology, Technical Medical Centre, University of Twente, Enschede, The Netherlands

**Keywords:** Microcirculation, Perfusion imaging, Phantoms (imaging), Reference standards

## Abstract

**Background:**

We aimed at reviewing design and realisation of perfusion/flow phantoms for validating quantitative perfusion imaging (PI) applications to encourage best practices.

**Methods:**

A systematic search was performed on the Scopus database for “perfusion”, “flow”, and “phantom”, limited to articles written in English published between January 1999 and December 2018. Information on phantom design, used PI and phantom applications was extracted.

**Results:**

Of 463 retrieved articles, 397 were rejected after abstract screening and 32 after full-text reading. The 37 accepted articles resulted to address PI simulation in brain (*n* = 11), myocardial (*n* = 8), liver (*n* = 2), tumour (*n* = 1), finger (*n* = 1), and non-specific tissue (*n* = 14), with diverse modalities: ultrasound (*n* = 11), computed tomography (*n* = 11), magnetic resonance imaging (*n* = 17), and positron emission tomography (*n* = 2). Three phantom designs were described: basic (*n* = 6), aligned capillary (*n* = 22), and tissue-filled (*n* = 12). Microvasculature and tissue perfusion were combined in one compartment (*n* = 23) or in two separated compartments (*n* = 17). With the only exception of one study, inter-compartmental fluid exchange could not be controlled. Nine studies compared phantom results with human or animal perfusion data. Only one commercially available perfusion phantom was identified.

**Conclusion:**

We provided insights into contemporary phantom approaches to PI, which can be used for ground truth evaluation of quantitative PI applications. Investigators are recommended to verify and validate whether assumptions underlying PI phantom modelling are justified for their intended phantom application.

## Key points

- Without a validated standard, interpretation of quantitative perfusion imaging can be inconclusive.
- Perfusion phantom studies contribute to ground truth evaluation.
- We systematically reviewed design and realisation of contemporary perfusion phantoms.
- Assessed phantom designs are diverse and limited to single tissue compartment models.
- Investigators are encouraged to adopt efforts on phantom validation, including verification with clinical data.

## Background

Perfusion imaging (PI) is a powerful method for assessing and monitoring tissue vascular status, and alterations therein. Hence, PI is generally aimed at distinguishing healthy from ischemic and infarcted tissue. PI applications cover various imaging modalities such as ultrasound, computed tomography (CT), positron emission tomography (PET), and magnetic resonance imaging (MRI) that can record perfusion parameters in a wide spread of tissues including brain, liver, and myocardial tissue. A distinction can be made between contrast-enhanced and non-contrast PI approaches. The pertinent signal intensity in tissue can be recorded as a function of time or after a time interval, called dynamic or static PI respectively. This systematic review focuses on dynamic PI, as this approach enables quantitative analysis and absolute quantification of perfusion. In dynamic PI, it is possible to construct mathematical models that fit image data with model parameters in order to explain observed response functions in tissue. For example, time-intensity curves highlight the distribution of contrast material into the tissue over time. Model outcomes include computation of absolute blood flow (BF), blood volume (BV), and/or mean transit times (MTTs) [1]. Multiple BF models of tissue perfusion exist, including model-based deconvolution, model-independent singular value decomposition and maximum upslope models [2]. These BF models are increasingly used in addition to standard semiquantitative analysis, as these show potential towards better accuracy and standardised assessment of perfusion measures [3–5].

Without a validated standard, interpretation of quantitative results can be challenging. Validation and/or calibration of absolute perfusion measures is required to ensure unrestricted and safe adoption in clinical routine [6–8]. Validation approaches include *in vivo*, *ex vivo*, *in vitro*, and *in silico* studies and combinations hereof. Each approach has advantages and disadvantages, and may differ in level of representativeness, controllability of variables, and practical applicability. Our focus was on *in vitro* studies, *i.e.,* physical phantom studies. Phantom studies contribute to ground truth evaluation of single aspects on quantitative PI applications in a simplified, though controlled, environment. Phantom studies also allow for the comparison and optimisation of imaging protocols and analysis methods. We hereby observe a shift from the use of static to dynamic perfusion phantoms (*i.e.,* with a flow circuit), as the latter enables in-depth evaluation of time-dependent variables.

In general, it can be challenging to translate findings from phantom studies into clinical practice. For example, it can be questionable whether certain choices and simplifications in perfusion phantom modelling are justified. Intra- and interdisciplinary knowledge sharing on phantom designs, experimental findings, and clinical implications can be used to substantiate this. Hence, this systematic review presents an overview on contemporary perfusion phantoms for evaluation of quantitative PI applications to encourage best quantitative practices.

## Methods

A systematic search on general and contemporary perfusion phantoms was conducted using Scopus database online, which includes MEDLINE and EMBASE. The query included “perfusion”, “flow”, and “phantom”. Inclusion was limited to English written articles and reviews published between January 1999 and December 2018.

Two investigators independently screened titles and abstracts (M.E.K. and M.J.W.G.), whereby *in vivo*, *ex vivo* and *in silico* related perfusion studies were excluded, even as non-related *in vitro* studies (*e.g.,* static and large-vessel phantoms). We hereby note that thermal and optical PI techniques fall outside the scope of this review. The same investigators performed full-text screening and analysis. Study inclusion required incorporation of microvascular flow simulation and we excluded single-vessel phantom studies. In addition, references were scrutinised on cross-references. Observer differences were resolved by discussion.

**Fig. 1:**
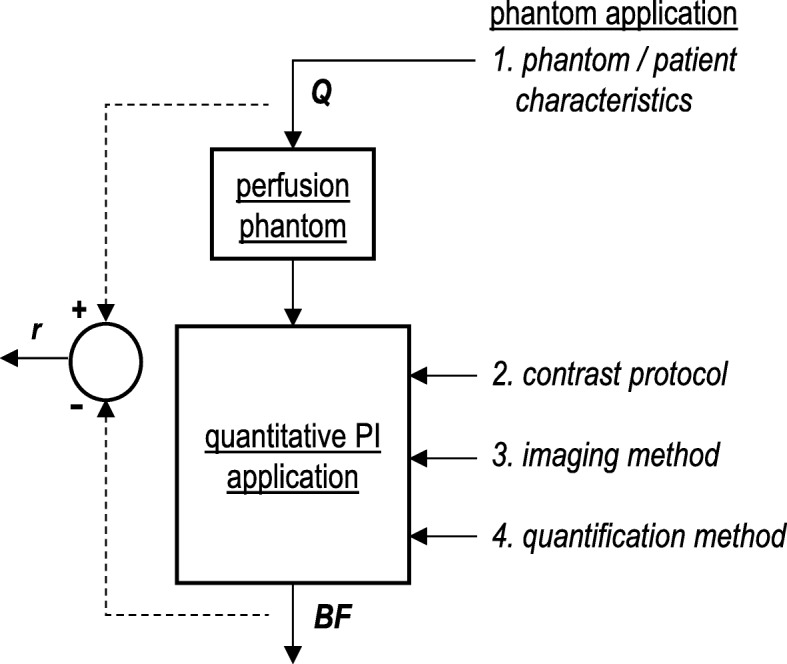
System representation of ground truth validation process of quantitative perfusion imaging (PI). The diverse input variables that might affect quantitative perfusion outcomes are shown on the right. *Q* serves as an example input variable and refers to set phantom flow in mL/min. *BF* is accordingly the computed blood flow in mL/min (system output), and *r* is the residual between both. The latter can be translated into a measure of accuracy. The figure summarises the central topics of this review paper

The perfusion phantom overview concerns three main aspects regarding ground truth evaluation of quantitative PI, as schematically depicted in Fig. 1. Details on perfusion phantom design, studied PI application and overall phantom application were extracted from each paper. We categorised phantom design features in terms of simulated anatomy, physiology, and pathology. Anatomy simulation lists information on the studied tissue type and surrounding tissue. Physiology simulation contains the used phantom configuration, the corresponding tissue-compartment model, the applied flow profile and range, and the simulation of motion (*e.g.,* breathing and cardiac motion). Pathology simulation indicates the presence of perfusion deficit simulation.

Extracted parameters for the studied PI application encounters the used contrast protocol, imaging system, and BF model. We also listed the studied input and output variables for the diverse phantom applications. Input variables were categorised as follows: (1) phantom/patient characteristics; (2) contrast protocol, if applicable; (3) imaging method; and (4) flow quantification method (see Fig. 1). Output variables included the following perfusion measures: arterial input function; tissue response function; MTT; BV; and BF. If mentioned by the authors, we listed published results on phantom performance, which describes the relation between the “ground truth” flow measure and the obtained quantitative PI outcomes. Finally, we documented in which studies phantom data are compared with human, animal, or mathematical data, and which phantoms are commercially available.

## Results

### Phantom data assessment

**Fig. 2:**
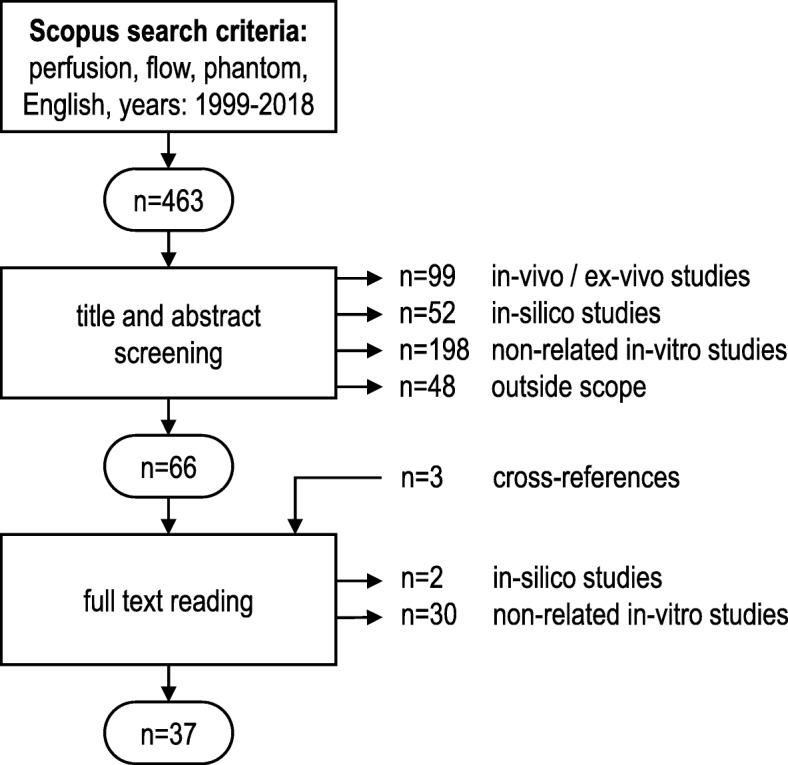
Flow chart of study selection process

We have retrieved 463 articles using Scopus, of which 397 were rejected after abstract screening and another 32 after full-text reading. The search resulted in 37 accepted articles including cross-references (Fig. 2). Tables 1, 2, 3, and 4 summarise our main findings on phantom designs and applications in diverse PI domains.

**Table 1:** Design and realisation of general perfusion phantoms in quantitative perfusion imaging (PI)

Publication	Phantom design			PI application						Phantom application							
1st author, year [reference]	Configuration (see Fig. 3)	Flow profile	Flow range	Motion simulation	Surrounding tissue simulation	Perfusion deficit simulation	Imaging modality	Contrast protocol	Blood flow model	Input variables	AIF	RF	MTT	BV	BF	Data comparison	Commercial
General phantoms																	
Andersen, 2000 [9]	1A	c	0.015–0.57 *C*				MRI		FAIR	1, 4		x			x		
Brauweiler, 2012 [10]	1A	p	180 *A*		x		CT	x		2, 3	x	x					
Li, 2002 [11]	1A, 2B	c	500–1300 *A*				US	x	MBD	1	x	x	x	x		M	
Peladeau-Pigeon, 2013 [12]	1B	c, p	210–450 *A*		x		MRI, CT	x	MBD (Fick, modif. Toft)	1–3	x	x			x	M	x
Driscoll, 2011 [13]	1B	p	150–270 *A*		x		CT	x		1–3	x	x					x
Kim, 2016 [14]	2B	c	0–2 *A*	x			US			1, 3		x					
Anderson, 2011 [15]	2B	c	50 *A*				MRI	x		1		x					
Meyer-Wiethe, 2005 [16]	2B	c	4.5–36 *A*				US	x	Replenishment	1, 3, 4		x					
Veltmann, 2002 [17]	2B	c	10–45 *A*				US	x	Replenishment	1, 2		x			x		
Kim, 2004 [18]	2B, 3A	p	0.09, 1.6–1.8 *C*				MRI		1-TCM	1, 3		x					
Lee, 2016 [19]	3A	c	0–3 *A*				MRI		DWI	1		x					
Chai, 2002 [20]	3A	c	50–300 *A*				MRI		ASL	1		x				H	
Potdevin, 2004 [21]	3A	p	2.6–10.4 *A*		x		US	x	Replenishment	1, 2		x	x			M	
Lucidarme, 2003 [22]	2B	p	100–400 *A*				US	x	Replenishment	1		x				A	

*c* Continuous, *p* Pulsatile, *A* in mL/min, *B* in mL/min/g, *C* in cm/s, *FAIR* Flow-sensitive alternating inversion recovery, *MBD* Model-based deconvolution, *1-TCM* Single tissue compartment model, *DWI* Diffusion weighted imaging, *ASL* Arterial spin labelling, *SVD* Singular value decomposition, *MSM* Maximum slope model, 1 = Phantom/patient characteristics, 2 = Contrast protocol, 3 = Imaging method, 4 = Flow quantification method, *AIF* Arterial input function, *RF* Response function, *MTT* Mean transit time, *BV* Blood volume, *BF* Blood flow, *H* Human, *A* Animal, *M* Mathematical

**Table 2:** Design and realisation of brain perfusion phantoms for quantitative perfusion imaging (PI)

Publication	Phantom design			PI application						Phantom application							
1st author, year [reference]	Configuration (see Fig. 3)	Flow profile	Flow range	Motion simulation	Surrounding tissue simulation	Perfusion deficit simulation	Imaging modality	Contrast protocol	Blood flow model	Input variables	AIF	RF	MTT	BV	BF	Data comparison	Commercial
Brain phantoms																	
Boese, 2013 [23]	1A	p	800 *A*			x	CT	x	MBD	1–3	x	x		x	x		
Hashimoto, 2018 [24]	2A	c	60 *A*		x		CT	x	SVD	2, 3			x	x	x	M	
Suzuki, 2017 [25]	2A	c	60 *A*		x		CT	x	SVD	3	x	x	x	x	x	M	
Noguchi, 2007 [26]	2A	c	0–2.16 *C*				MRI		ASL	1		x			x		
Wang, 2010 [27]	2B	c	45–180 *A*				MRI		ASL	1		x			x	M, H	
Cangür, 2004 [28]	2B	c	1.8–21.6 *A*		x		US	x		1		x					
Klotz, 1999 [29]	2B	c	50–140 *A*		x		CT	x	MSM	1	x	x			x	H	
Claasse, 2001 [30]	2B	p	180–540 *A*				US	x	MBD	1, 2		x	x			A	
Mathys, 2012 [31]	3A	c	200–600 *A*		x		CT	x	SVD, MSM	1–4	x	x		x	x		
Ebrahimi, 2010 [32]	3A	c	012–1.2 *A*				MRI	x	SVD	1	x	x	x	x	x	M	
Ohno, 2017 [33]	3B	p	240–480 *A*				MRI		ASL	1		x			x		

*c* Continuous, *p* Pulsatile, *A* in mL/min, *B* in mL/min/g, *C* in cm/s, *FAIR* Flow-sensitive alternating inversion recovery, *MBD* Model-based deconvolution, *1-TCM* Single tissue compartment model, *DWI* Diffusion weighted imaging, *ASL* Arterial spin labelling, *SVD* Singular value decomposition, *MSM* Maximum slope model, 1 = Phantom/patient characteristics, 2 = Contrast protocol, 3 = Imaging method, 4 = Flow quantification method, *AIF* Arterial input function, *RF* Response function, *MTT* Mean transit time, *BV* Blood volume, *BF* Blood flow, *H* Human, *A* Animal, *M* Mathematical

**Table 3:** Design and realisation of general myocardial phantoms for quantitative perfusion imaging (PI)

Publication	Phantom design			PI application						Phantom application							
1st author, year [reference]	Configuration (see Fig. 3)	Flow profile	Flow range	Motion simulation	Surrounding tissue simulation	Perfusion deficit simulation	Imaging modality	Contrast protocol	Blood flow model	Input variables	AIF	RF	MTT	BV	BF	Data comparison	Commercial
Myocardial phantoms																	
Zarinabad, 2014 [34]	2A	c	1–5 *B*				MRI	x	MBD (Fermi)	1, 4	x	x			x	M, H	
Chiribiri 2013 [8]	2A	c	1–10 *B*				MRI	x		1, 2	x	x					
Zarinabad, 2012 [35]	2A	c	1–5 *B*				MRI	x	MBD (Fermi), SVD	1, 3, 4	x	x			x	M, H	
O’Doherty, 2017 [36]	2A	c	3 *B*				PET,MRI	x	1-TCM	2, 3	x	x			x		
O’Doherty, 2017 [37]	2A	c	1–5 *B*				PET,MRI	x	1-TCM	1, 3	x	x			x		
Otton, 2013 [38]	2A	c	2–4 *B*				MR,CT	x		1, 3	x	x					
Ressner, 2006 [39]	3A	c	5–10 *C*	x			US	x		1, 2		x			x	H	
Ziemer, 2015 [40]	3A	p	0.96–2.49 *B*		x		CT	x	MSM	1, 4	x	x			x		

*c* Continuous, *p* Pulsatile, *A* in mL/min, *B* in mL/min/g, *C* in cm/s, *FAIR* Flow-sensitive alternating inversion recovery, *MBD* Model-based deconvolution, *1-TCM* Single tissue compartment model, *DWI* Diffusion weighted imaging, *ASL* Arterial spin labelling, *SVD* Singular value decomposition, *MSM* Maximum slope model, 1 = Phantom/patient characteristics, 2 = Contrast protocol, 3 = Imaging method, 4 = Flow quantification method, *AIF* Arterial input function, *RF* Response function, *MTT* Mean transit time, *BV* Blood volume, *BF* Blood flow, *H* Human, *A* Animal, *M* Mathematical

**Table 4:** Design and realisation of finger, liver, and tumour perfusion phantoms for quantitative perfusion imaging (PI)

Publication	Phantom design			PI application						Phantom application							
1st author, year [reference]	Configuration (see Fig. 3)	Flow profile	Flow range	Motion simulation	Surrounding tissue simulation	Perfusion deficit simulation	Imaging modality	Contrast protocol	Blood flow model	Input variables	AIF	RF	MTT	BV	BF	Data comparison	Commercial
Finger phantom																	
Sakano, 2015 [41]	2B	c	6–30 *A*				US	x		1, 3		x					
Liver phantoms																	
Gauthier, 2011 [42]	2B	c	130 *A*				US	x		3		x	x			H	
Low, 2018 [43]	3A	-	20.5 *A*				CT	x		1							
Tumour phantom																	
Cho, 2012 [44]	3A,3B	p	-				MRI		DWI	1, 4		x					

*c* Continuous, *p* Pulsatile, *A* in mL/min, *B* in mL/min/g, *C* in cm/s, *FAIR* Flow-sensitive alternating inversion recovery, *MBD* Model-based deconvolution, *1-TCM* Single tissue compartment model, *DWI* Diffusion weighted imaging, *ASL* Arterial spin labelling, *SVD* Singular value decomposition, *MSM* Maximum slope model, 1 = Phantom/patient characteristics, 2 = Contrast protocol, 3 = Imaging method, 4 = Flow quantification method, *AIF* Arterial input function, *RF* Response function, *MTT* Mean transit time, *BV* Blood volume, *BF* Blood flow, *H* Human, *A* Animal, *M* Mathematical

### Phantom design

**Fig. 3:**
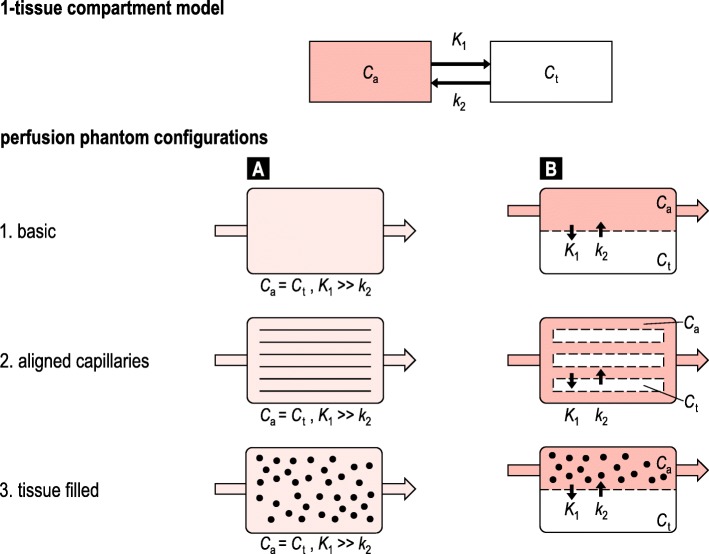
Schematic representation of the 1-tissue compartment model and six derived phantom configurations. A distinction is made between three phantom types: basic, aligned capillaries and tissue filled (black spheres). Moreover, the microvasculature and tissue can be simulated as one combined (**a**) or two separated volumes (**b**) (*e.g.,* via a porous membrane). *C*_*p*_ and *C*_*t*_ represent the concentration of the compound of interest (is being imaged) in the simulated blood plasma and tissue, respectively. *K*_1_ and *k*_2_ comprise the two transfer coefficients. Formation of in- and outgoing flow (arrow) and compartment flow varies per individual phantom design

Anatomically, the phantoms simulate perfusion of various tissue types, including organ specific tissue (brain, *n* = 11 articles; myocardial, *n* = 8; liver, *n* = 2; tumour, *n* = 1; finger, *n* = 1) and non-specific tissue (*n* = 14). Several phantoms additionally mimic surrounding tissue (Tables 1, 2, 3, and 4). All phantoms comprise a simplified “physiologic” model of perfusion that can be translated into a single tissue compartment model. Figure 3 schematically illustrates the basics of six distinguished phantom configurations, which specify three phantom types: basic (*n* = 6 articles); aligned capillaries (*n* = 22); and tissue filled (*n* = 12). The observed phantom designs simulate the microvasculature and tissue as one combined volume (*n* = 23 articles) or two physically separated volumes (*n* = 17) (*e.g.,* via a semipermeable membrane). Note that papers can present more than one phantom, and phantom designs may slightly differ from the schematic representations.

**Fig. 4:**
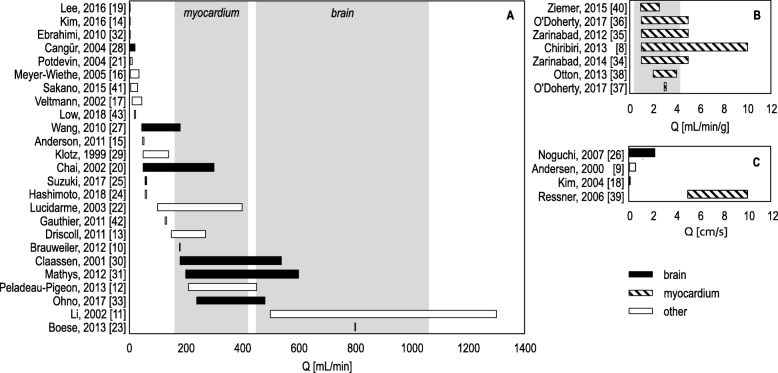
Overview of used flow ranges and units in assessed perfusion phantom studies. (**a**) shows the studied flow ranges in mL/min, (**b**) in mL/min/g, and (**c**) in cm/s. The grey blocks represent physiological flow ranges for brain and myocardial tissue [45, 46]

*Basic phantoms* generally consist of a single volume with ingoing and outgoing tubes, disregarding physiological simulation of microcirculation and tissue. In *capillary phantoms*, the microvasculature is simulated as a volume filled with unidirectional aligned hollow fibres or straws (*e.g.,* a dialysis cartridge). The amount, diameter, and permeability of these fibres vary. *Tissue-filled* phantoms incorporate tissue-mimicking material inside the volume, which subsequently leads to formation of a “microvasculature”. Used materials include sponge [20, 21, 33, 44], (micro)beads [19, 31, 40], gel [18, 39], and printed microchannels [32, 43]. Remarkably, in most studies, fluid exchange between simulated microvasculature and tissue (*i.e.,* transfer rates *K*_*1*_ and *k*_*2*_) was uncontrollable, except for the study performed by Ohno et al. [33]. In this study, the compliance of the capacitor space could be altered to control *k*_*2*_ to some extent. Low et al. [43] and Ebrahim et al. [32] have mathematically simulated the desired phantom flow configuration, before printing the microchannels. However, these models did not simulate fluid exchange between microvasculature and tissue. Continuous flow was applied in 26 phantom studies and pulsatile/peristaltic flow in 11 phantom studies. Flow settings vary per study and target organ and are presented in three different units (Tables 1, 2, 3, and 4). In case of brain and myocardial perfusion phantom modelling, flow experiments do not always cover the whole physiological range (Fig. 4). In addition, we observed two phantom studies that incorporated clutter motion (*i.e.,* small periodic motion), but no studies included breathing or cardiac motion (Tables 1, 2, 3, and 4). Regional perfusion deficit simulation (pathology) was only executed by Boese et al. [23]. Several studies mimicked some sort of global perfusion deficits by reducing the total flow or perfusion rate.

### Studied PI applications

Tables 1, 2, 3, and 4 depict 17 studies focusing on MRI, 11 on ultrasound imaging, 11 on CT, and 2 on PET; 4 studies presented a direct comparison of MRI with PET or CT. A contrast-enhanced protocol was used in 28 studies. The used BF model for perfusion quantification varies per imaging modality and contrast protocol.

### Phantom applications

Variables related to phantom/patient characteristics (*n* = 32), contrast protocol (*n* = 12), imaging method (*n* = 16), and quantification method (*n* = 7) were studied in relation to various quantitative perfusion measures (Table 1). Most papers describe the influence of flow settings on quantitative perfusion outcomes, followed by variation in contrast volume and acquisition protocol. Several studies compare outcomes to human/patient data (*n* = 7), animal data (*n* = 2), and mathematical simulations (*n* = 9) (Table 1). In addition, we have identified one commercially available perfusion phantom that is described by Driscoll et al. [13] and applied by Peladeau-Pigeon et al. [12]. The relation between the “ground truth” flow measure and quantitative PI outcomes is summarised in Table 5. Remarkable is the diversity in used measures of perfusion and comparison (*e.g.,* absolute errors, correlations statistics).

**Table 5:** Design and realisation of brain perfusion phantoms for quantitative perfusion imaging (PI)

1st author, year [reference]	Perfusion measure(s)	Phantom performance	Q
Direct comparison with Q			
Klotz, 1999 [29]	BF	*r* = 0.990	50–140 mL/min
Wang, 2010 [27]	BF	*r* > 0.834	45–180 mL/min
Mathys, 2012 [31]	BF	*r* = 0.995	200–600 mL/min
Peladeau-Pigeon, 2013 [12]	BF	*r* = 0.992	210–450 mL/min
Ohno, 2017 [33]	BF	*r* > 0.90	240–480 mL/min
Ziemer, 2015 [40]	BF	*r* = 0.98	0.96–2.49 mL/g/min
O’Doherty, 2017 [36]	BF	*r* = 0.99	1–5 mL/g/min
Andersen, 2000 [9]	BF	*ε* ≈ 0.015 ± 0.03 cm/s *ε* ≈ 0.001 ± 0.03 cm/s	0.015 ± 0.002 cm/s 0.570 ± 0.003 cm/s
Ressner, 2006 [39]	BF	*ε* > 40% *ε* < 20%	1–3 cm/s 5–7 cm/s
Zarinabad, 2012 [35]	BF	*ε* = 0.007 ± 0.002 mL/g/min *ε* = 0.23 ± 0.26 ml/g/min	0.5 mL/g/min 5 mL/g/min
Zarinabad, 2014 [34]	BF	*ε* < 0.03 mL/g/min *ε* < 0.05 ml/g/min	2.5–5 mL/g/min 1–2.5 mL/g/min
Suzuki, 2017 [25]	BF	*ε* ≈ 0.0589 ± 0.0108 mL/g/min	0.1684 mL/g/min
Hashimoto, 2018 [24]	BF	*ε* ≈ 0.0446 ± 0.0130 mL/g/min	0.1684 mL/g/min
Ebrahimi, 2019 [32]	BF	BF/Q > 0.6	0.12–1.2 mL/min
Indirect comparison with Q			
Veltmann, 2002 [17]	*r*_kin_	*r* > 0.984, *χ*^2^ < 0.019	10–45 mL/min
Chai, 2002 [20]	∆SI ratio	*r* = 0.995	50–300 mL/min
Cangür, 2004 [28]	TTP PSI AUC PG FWHM	*r* = -0.964 *r* = 0.683 *r* = 0.668 *r* = 0.907 *r* = -0.63	1.8–21.6 mL/min
Myer-Wiethe, 2005 [16]	∆SI	*r* = 0.99	4.5–36 mL/min
Lee, 2016 [19]	*f*_p_	*r* > 0.838	1–3 mL/min
O’Doherty, 2017 [36]	SI	*r* = 0.99 *r* = 0.99	1–5 mL/g/min (MRI) 1.2–5.1 mL/g/min (MRI *vs* PET)
Kim, 2016 [14]	AUC	Efficiency <50%	0.1–2.0 mL/min
Claassen, 2001 [30]	AUC, PSI, MTT	No clear correlation with Q	

Phantom performance is predominantly listed in correlation statistics (*r*, *χ*^2^) and absolute errors (*ε*). A distinction is made between direct and indirect comparison with a “ground truth” flow measure (Q), which consists of theoretical or experimental values. *BF* Blood flow, *TTP* Time to peak, *MTT* Mean transit time, *AUC* Area under the curve, (*P)SI* Peak signal intensity, *f*_p_ Perfusion fraction, *r*_kin_ Replenishment kinetics, *FWHM* Full width at half maximum, *PG* Positive gradient

## Discussion

A systematic search of the literature (from 1999 to 2018) was performed on contemporary perfusion phantoms. Detailed information was provided on three main aspects for ground truth evaluation of quantitative PI applications. We have elaborated on thirty-seven phantom designs, whereby focusing on anatomy, physiology and pathology simulation. In addition, we have listed the imaging system, contrast protocol and BF model for the studied PI applications. Finally, we have documented for each phantom application the investigated input and output variables, data comparison efforts and commercial availability. Hence, this review presents as main result an overview on perfusion phantom approaches and emphasises on the choices and simplifications in phantom design and realisation.

Although perfusion phantom modelling involves various tissues and applies to divers PI applications, we observe similarities in overall phantom designs and configurations. These configurations can be categorised in three types (6/40 basic, 22/40 capillary, and 12/40 tissue filled) and two representations of microvasculature and tissue (23/40 as one combined and 17/40 as two separated compartments). Differences in these six phantom configurations are reflected in the resulting flow dynamics, *e.g.,* how a contrast material is distributed and how long it stays inside the simulated organ tissue. None of the assessed phantoms could control inter-compartmental fluid exchange. Ideally, one would be able to fine-tune the exact flow dynamics in perfusion phantom modelling to achieve patient realistic (and contrast material specific) response function simulation. The required level of representativeness depends on the intended analyses, being closely related to the input parameters and boundary conditions of the BF model used. Since all assessed phantoms are limited to single tissue compartment models, phantom validation of higher order BF models should be performed with caution. It is generally important to verify whether assumptions in phantom modelling are justified for the intended phantom application. This also concerns decisions regarding motion, pulsatile flow and perfusion deficit simulation. For example, in myocardial perfusion modelling it could be relevant to incorporate respiratory and cardiac motion for certain analyses [47, 48], while for other tissues “motion” could be disregarded more easily.

The need for standardisation and validation of (quantitative) PI applications is widely recognised [49, 50]. Perfusion phantom studies contribute to this endeavour, since these studies enable direct comparison between imaging systems and protocols. We only observed one commercial perfusion phantom in our search result. We foresee an increased clinical impact when phantoms become validated and widely available. In our opinion, phantom validation efforts are sometimes reported insufficiently and ambiguously. The concept of *phantom validation* can be difficult, since it is application-dependent and prone to subjectivity. The latter becomes apparent in the use of the words “considered”, “reasonable”, and “acceptable” (by whom, to whom, according to which criteria?) [51]. We therefore suggest to use Sargent’s theory on model verification and validation [52]. Van Meurs’ interpretation of this theory, including a practical checklist, is also applicable to physical, biomedical models (in adjusted form) [51]. For example, according to the checklist, investigators should verify whether the applied flow range covers the full physiological range. Our results (see Fig. 4) show great diversity in measured flow ranges. In addition, investigators are advised to consult physiologists and clinicians along the process, and compare findings with clinical data. In nine studies, phantom data are indeed compared with human or animal perfusion data (see Table 1).

When analysing phantom results, we noticed that investigators use different measures to evaluate quantitative PI outcomes, which hampers comparability (see Table 5). Some investigators express the relation between quantitative PI outcomes and the “ground truth” flow in correlation statistics or plots and others in absolute errors. Due to the diversity in outcome measures, applied flow ranges, and amount of measurements carried out, interpretation of these results should be handled with caution. A uniform, unambiguous measure to evaluate both phantom validity and the accuracy and precision of quantitative PI outcomes is desired.

This study has limitations. Our search was limited to articles published between 1999 and 2018, yet we are aware that the development and use of perfusion phantoms date further back. Contemporary studies build on these designs, which makes it relevant to elaborate on perfusion phantom experiments in advanced PI systems. Furthermore, we have decided to leave out detailed information on phantom design and fabrication (*e.g.,* material choices and dimensions), since this information can be found in the appropriate references. Besides, phantom manufacturing is highly subject to change. We expect to see more three-dimensional printed perfusion phantoms in the coming years [43, 53, 54].

In conclusion, this systematic review provided insights into contemporary perfusion phantom approaches, which can be used for ground truth evaluation of quantitative PI applications. It is desirable to indicate an unambiguous measure for phantom validity. Furthermore, investigators in the field are recommended to perform measurements in the full physiological flow range, consult physiologists and clinicians along the process, and compare findings with clinical data. In this way, one can verify and validate whether made choices and simplifications in perfusion phantom modelling are justified for the intended application, hence increasing clinical impact.

## Data Availability

All data generated or analysed during this study are included in this published article.

## Abbreviations

BF: Blood flow
BV: Blood volume
CT: Computed tomography
MRI: Magnetic resonance imaging
MTT: Mean transit time
PET: Positron emission tomography
PI: Perfusion imaging
*Q*: “Ground truth” flow measure
